# Implementation Evaluation of a Cluster Randomized Controlled Trial to Promote the Use of Respiratory Protective Equipment Among Migrant Workers Exposed to Organic Solvents in Small and Medium-Sized Enterprises

**DOI:** 10.3389/fpubh.2022.772632

**Published:** 2022-07-11

**Authors:** Chuangpeng Lin, Tongyang Li, Guanyang Zou, Xudong Li, Li Ling, Wen Chen

**Affiliations:** ^1^Department of Medical Statistics, School of Public Health, Sun Yat-sen University, Guangzhou, China; ^2^Center for Migrant Health Policy, Sun Yat-sen University, Guangzhou, China; ^3^School of Public Health and Management, Guangzhou University of Chinese Medicine, Guangzhou, China; ^4^Guangdong Prevention and Treatment Center for Occupational Diseases, Guangzhou, China

**Keywords:** implementation evaluation, occupational safety and health, migrant workers, small and medium-sized enterprises, RE-AIM framework

## Abstract

**Background:**

While the effectiveness of several occupational healthcare interventions has been demonstrated, successful implementation of such programs among internal migrant workers (IMWs) in small and medium-sized enterprises (SMEs) has been limited. This study aimed to evaluate the implementation of a three-arm cluster randomized controlled trial promoting respiratory protective equipment (RPE) use among IMWs exposed to organic solvents in SMEs and to assess the association between participants' compliance and effectiveness of intervention.

**Methods:**

A total of 60 SMEs were randomly allocated to a low- or high-intensive intervention group, or a control group that did not receive any intervention. The low-intensive intervention group was subjected to both traditional and mHealth occupational health education. The high-intensive intervention group was subjected to the low-intensive group activities and peer education. The Reach, Effectiveness, Adoption, Implementation, and Maintenance (RE-AIM) framework was used to guide implementation evaluation of this 6-months intervention. Generalized linear mixed models (GLMMs) were used to evaluate the effects of participants' compliance with the intervention on the primary outcomes, regarding the appropriate use of RPE.

**Results:**

Of 4,527 potentially eligible participants, 1,211 individuals were enrolled, with a reach rate of 26.8%. Sixty of the 66 SMEs approached (90.9%) SMEs adopted the intervention. Fidelity to traditional education (100.0%) and mHealth intervention (97.5%) was higher than fidelity to peer education (20.0%). Peer leaders cited inconvenient time and unfamiliarity with peers as two major barriers to delivering peer education. Compared with the control group, IMWs who complied with the interventions in both groups were more likely to wear RPE appropriately [low-intensive group: adjusted odds ratio (aOR) = 2.58, 95% confidence interval (CI): 1.56–4.28; high-intensive group: aOR = 7.52, 95% CI: 3.72–15.23]. Most participants (95.8%) were satisfied with the program and 93.1% stated that they would maintain the use of RPE in the future.

**Conclusions:**

A multi-component occupational health intervention to promote the use of RPE among IMWs in SMEs was feasible and acceptable. Peer education had great potential to enhance the occupational health behavior of IMWs, and thus strategies to improve participants' adherence to this component warrant further investigation.

**Clinical Trial Registration:**

http://www.chictr.org.cn, identifier: ChiCTR-IOR-15006929.

## Introduction

The World Health Organization recently highlighted that the occupational safety and health (OSH) of workers is a major challenge in the implementation of sustainable development initiatives and workplace health promotions worldwide (1). According to the International Labor Organization, approximately 2.3 million people die from work-related diseases and injuries each year (2). The annual number of occupational diseases diagnosed in China increased from 12,212 in 2005 to 19,428 in 2019, with an average annual growth rate of 3.37% (3, 4). The Chinese Government has prioritized occupational health as one of 15 major health projects (5), and the National Health Commission (NHC) has unveiled new strategies to reduce the incidence of occupational diseases, focusing on major industries, occupational hazards, and victims (6).

It was estimated that, in China, approximately 100 million internal migrant workers (IMWs) were exposed to occupational hazards in 2018 (7), while organic solvents were the second most common occupational hazards after pneumoconiosis. In light of this, we conducted the first clustered randomized controlled trial (CRCT) to promote the use of respiratory protective equipment (RPE) among IMWs exposed to organic solvents from small and medium-sized enterprises (SMEs) in China (8). The CRCT was a multifactorial complex intervention encompassing three main components: (1) traditional occupational health education, (2) mobile health (mHealth) intervention, and (3) peer education. Effectiveness evaluation of a previous multi-component intervention (9) revealed that participants' adherence to the prescribed peer education was not ideal, but it was a key factor influencing outcomes, indicating a need to explore the process of intervention implementation and its impact on effectiveness.

Implementation evaluation could help researchers better understand the implementation of an intervention and the facilitators of and barriers to intervention delivery (10, 11). When used in combination with effectiveness evaluation, implementation can provide an evidence base for the wide-scale implementation, thereby enhancing the potential population-based impact and facilitating the generalizability and application of such programs to similar contexts (12). However, to the best of our knowledge, research on the implementation evaluation of effective behavioral interventions for IMWs in SMEs is lacking. Similarly, although an increasing number of occupational health promotion program have demonstrated effectiveness in reducing work-related disease risk in Canada (13), Australia (14), America (15) and the United Kingdom (16), few studies have paid attention to understanding the implementation of the interventions.

Since the 1990s, rapid growth in public health promotion programs has led to an increasing emphasis on the process of program implementation and the emergence of a variety of guidelines for implementation evaluation. The RE-AIM framework (17) was one of the most widely used theoretical frameworks for understanding the effectiveness of programs implemented in real-world settings (18, 19). Glasgow proposed that process evaluations of health promotion interventions should be assessed from five dimensions: Reach, Effectiveness, Adoption, Implementation, and Maintenance (18). In this study, we set two aims: (1) to evaluate the implementation of the aforementioned multi-component occupational health behavioral intervention using Glasgow's RE-AIM framework, including the program reach, program effectiveness, intervention fidelity, barriers to intervention delivery, participants' compliance, participants' satisfaction, and willingness to maintain the strategies learned from during the program; and (2) to assess the association of participants' compliance with intervention and effectiveness of the intervention.

## Methods

### Study Design, Setting, and Participants

Details of the study protocol, which was prepared according to CONSORT guidelines, have been published elsewhere (8). Briefly, we conducted a three-arm CRCT at both the enterprise and worker levels from August 2015 to January 2016. This trial aimed to assess the effectiveness of a 6-months multi-component occupational health behavioral intervention for promoting the appropriate use of RPE. The Research Ethics Committee of the School of Public Health, Sun Yat-sen University approved this trial, which has been registered in the Chinese Clinical Trial Register (ChiCTR-IOR-15006929).

Sixty SMEs were randomly selected by the researchers from a list of 861 eligible SMEs which was provided by the Administration of Work Safety in Baiyun district, Guangzhou city, China; this administration is responsible for OSH in the district. Baiyun district is inhabited by a significant number of migrants and is the most industrially developed area in Guangzhou. The inclusion criteria for the enterprises selected were: (1) 20 to 1,000 workers and an annual turnover of 3 to 400 million Chinese Yuan, according to the definition developed by the Ministry of Industry and Information Technology (20); (2) work involving the use of organic solvents; (3) provision of RPE to workers, including disposable masks, half-face masks and full-face masks. The 60 SMEs were randomly allocated to equivalent numbers of the low-intensive intervention (*n* = 20), high-intensive intervention (*n* = 20), and control (*n* = 20) groups.

All eligible IMWs at the selected SMEs were approached by their managers and invited to participate in the trial. Workers without a local registered permanent residence and who were employed working on a production line involving exposure to organic solvents were eligible for inclusion. First-line production team leaders and those unable to complete the questionnaires due to illiteracy were excluded. All study participants provided written informed consent.

### Interventions

Participants in the control group were not offered any intervention. Participants in the two intervention groups received a complex multicomponent occupational health behavioral intervention at both the enterprise and participant levels.

The low-intensive intervention programs comprised two elements: traditional occupational health education and mHealth intervention. Traditional occupational health education included a 1-h occupational health lecture for enterprise managers and IMWs during the first week of the intervention, with assistance by the Guangdong Prevention and Treatment Center for Occupational Diseases. The lectures were intended to help enterprises strengthen their knowledge of OSH responsibilities and increase IMWs' understanding of the hazards of organic solvents exposure, the benefits of RPE, and the proper selection and maintenance of RPE. At baseline and the third month of the intervention, occupational health-related brochures were distributed to IMWs, and posters were hung in the workspace at every participating enterprise. The mHealth intervention included: image- and video- based educational resources about organic solvent hazards, personal occupational protection, and occupational health laws and regulations, which were delivered to the IMWs twice per week *via* an instant message app, such as WeChat or QQ. Online quizzes with awards were used to promote increased participation in the third and sixth months of the intervention.

IMWs in the high-intensive group received the same intervention as the low-intensive group, but also attended peer education sessions delivered by peer leaders. The peer education group was composed of 8–15 IMWs, with one volunteering as a peer leader. Peer leaders received two 1-h sessions of training provided by the research team, and delivered peer education sessions to their peers per month (six sessions in total) according to a 33-page peer education manual formulated by the research team. We conducted on-line supervision and assessments of peer leaders and awarded 50–100 Chinese Yuan (8–16 USD) each month to the top five best performing to incentivize engagement with the program.

### Implementation Evaluation

Implementation outcomes were evaluated using the RE-AIM framework (17). We identified 27 specific process evaluation indicators for our program (Table 1) from the 34 evaluation criteria originally proposed by Glasgow, which vary considerably across different programs due to variable program design and implementation (18).

**Table 1:** Summary of process evaluation dimensions, indicators, and data sources.

**Dimensions**	**Definitions**	**Indicators**	**Data sources**
Reach	The absolute number, proportion, and representativeness of individuals who were willing to participate in a given intervention	**Participant level:** Number of participants approached in the target population Number of and reasons for exclusions Number of eligible participants who were offered participation Percentage participation of all potential participants Percentage drop-out Characteristics of participants Description of recruitment procedure	Recruitment records Participants surveys
Effectiveness	The impact of the intervention on important outcomes as well as the heterogeneity of the effects	**Participant level:** Impact of the intervention on primary outcome Impact of the intervention on secondary outcomes Robustness across subgroups Differential effects by participant characteristics or treatments	Participants surveys
Adoption	The absolute number, proportion, and representativeness of settings and intervention agents (people who deliver the program) who were willing to initiate a program	**Enterprise level:** Number of eligible enterprises Number of enterprises invited to participate Number and proportion of enterprises that agreed to participate Proportion of and reasons for excluded organizations Characteristics of participating enterprises **Intervention deliverer level:** Number of staff assisting the implementation of the trial Number of peer-leaders delivering peer education sessions	Recruitment records Enterprises' investigations Peer leaders surveys
Implementation	The fidelity of various elements of an intervention's protocol, and clients' reception of the intervention	Enterprise level: Extent to which the interventions were delivered as intended Consistency of implementation across enterprises **Peer leader level:** Number and proportion of peer education sessions organized Methods of delivery of the peer education sessions Facilitators of and barriers to peer education sessions delivery **Participant level:** Participants' compliance with the intervention Participants' satisfaction with the program	Researchers' observations, Peer leaders' progress reports, Peer leaders surveys, Participants surveys
Maintenance	The extent to which a program became part of routine organizational practices and the long-term effects on outcomes 6 months or more post-intervention	**Peer leader level:** Willingness to supervise their work mates' use of RPE **Participant level:** Willingness to maintain RPE use in the future	Peer leaders surveys Participants surveys

The Reach dimension included seven indicators covering different aspects of recruitment process of participants, and the demographic characteristics of enrolled participants.

The Effectiveness dimension included four indicators measuring the impacts of the intervention on primary and secondary outcomes among overall participants and in different subgroups. The primary outcome was increased appropriate use of RPE, which was defined as an increase in IMWs who always wore the appropriate type of RPE at work during the last week of assessment, according to the guideline on the use of RPE against hazards from organic solvents promulgated by the NHC (21). Secondary outcomes included occupational health knowledge, attitude toward occupational health, and participation in occupational health check-ups during the program (see Supplementary Table 1 for measures of secondary outcomes) (9).

The Adoption dimension was assessed at two levels, namely the enterprise level and the deliverer level. Enterprise-level adoption included five indicators referring to the number, proportion, and characteristics of enterprises that adopted the program. Deliverer-level adoption measured the numbers of intervention deliverers, including staff assisting implementation of the trial and peer leaders.

The Implementation dimension was also assessed at two levels. Setting-level implementation was defined as the fidelity of various intervention components and the implementation details, including the methods and frequencies. Fidelity was measured as the extent to which the intervention was implemented perfectly as planned, and the consistency of implementation across enterprises. The methods and frequencies of sessions delivery by peer leaders, and the facilitators of and barriers to delivery were also evaluated. Participant-level implementation was defined as the participants' reception of the program, as reflected in compliance and satisfaction. In our study, participants' compliance was measured by six items: (1) participated in occupational health lectures; (2) browsed occupational health- related posters; (3) joined the WeChat or QQ groups of the program; (4) followed the WeChat official account of the program; (5) read the messages provided via the instant message apps; (6) the number of peer education sessions attended. The scores of items (1) and (2) (0 = no, 1 = yes) represented compliance with traditional occupational health education; and the scores of items (3), (4) and (5) (0 = no, 1 = yes) represented compliance with mHealth intervention; and the score of item (6) (0 = 0 session, 1 = 1–3 sessions, 2 = 4–6 sessions) represented compliance with peer education, with higher scores indicating greater compliance. Overall compliance in the low-intensive intervention group was defined as a total score of 3 or more (ranging from 0 to 5) and at least 1 point each for both traditional occupational health education and mHealth intervention. Overall compliance in the high-intensive intervention group was defined as a total score of 4 or more (ranging from 0 to 7) and at least 1 point each for traditional occupational health education, mHealth intervention, and peer education (Supplementary Table 1).

Due to limited time and fixed research funding, we did not assess maintenance of primary and secondary outcomes post-intervention. However, sustainability was addressed in the self-reported questionnaires administered to migrant workers at 6 months, which asked about the willingness of peer leaders to supervise their workmates using RPE and willingness of IMWs to maintain proper use of RPE in the future.

The corresponding implementation evaluation data were collected using a self-reported questionnaire surveys, recruitment records, enterprises investigations, peer leaders surveys, and researchers' observations at baseline and at the 3- and 6-months follow-ups. Additionally, the peer leaders were required to submit monthly progress reports containing a standard checklist that documented the implementation of peer education, and photos showing attendance of the peer education sessions. Process evaluation was undertaken at all stages of the intervention. Study staff checked all returned data and documents for completeness and inconsistencies to ensure the quality of the implementation data.

### Statistical Analysis

Descriptive statistics were used to describe the program characteristics and implementation outcomes based on the five dimensions of the RE-AIM framework. Continuous variables are summarized using means and standard deviations (SD). Categorical variables are presented as frequencies and percentages. We summarized baseline variables for the low- and high-intensive intervention and control groups separately. Additionally, we calculated the percentages of compliance with three intervention components of the program.

The chi-squared test was used to compare participants' compliance with the interventions between low- and high-intensive intervention groups. A generalized linear mixed model (GLMM) with a logistic link, a random effect with a compound symmetry correlation matrix to account for enterprise-level clustering, and a nested random effect to account for repeated measures within IMWs, was used to estimate the effect of compliance on the primary outcome. Based on the previous research (22, 23), the covariates included age, gender, marital status, education, duration of migration, duration of employment at current position, duration of employment at current workplace, weekly working hours, interpersonal support, social model, enterprise scale, and occupational health service provision of the enterprise. We adjusted all estimates for these baseline covariates. The adjusted odds ratios (aOR) and 95% confidence intervals (CI) of compliance in each intervention group compared with the control group were calculated to assess the impact of participants' compliance on the primary outcome.

To test the robustness and heterogeneity of effectiveness across subgroups, we performed subgroup analyses according to age (≤ 28, 29–44, or 45–65 years); gender (male or female); marital status (married or unmarried); education (primary school, secondary school, or high school and above); duration of migration (> 10 or ≤ 10 years); having dermatitis, eczema or conjunctivitis in the past month (yes or no); having a cold or other respiratory disease in the past month (yes or no); and feeling depressed or anxious in the past month (yes or no). We analyzed subgroups using statistical tests of interaction while adjusting for other covariates at baseline. Two-tailed tests with a significance level of *p* < 0.05 were applied to all analyses using IBM SPSS Statistics 26.0 (IBM Corp., Armonk, NY, United States). R 4.0.5 (24) was used to create a forest plot for subgroup analyses.

## Results

### Reach

**Figure 1:**
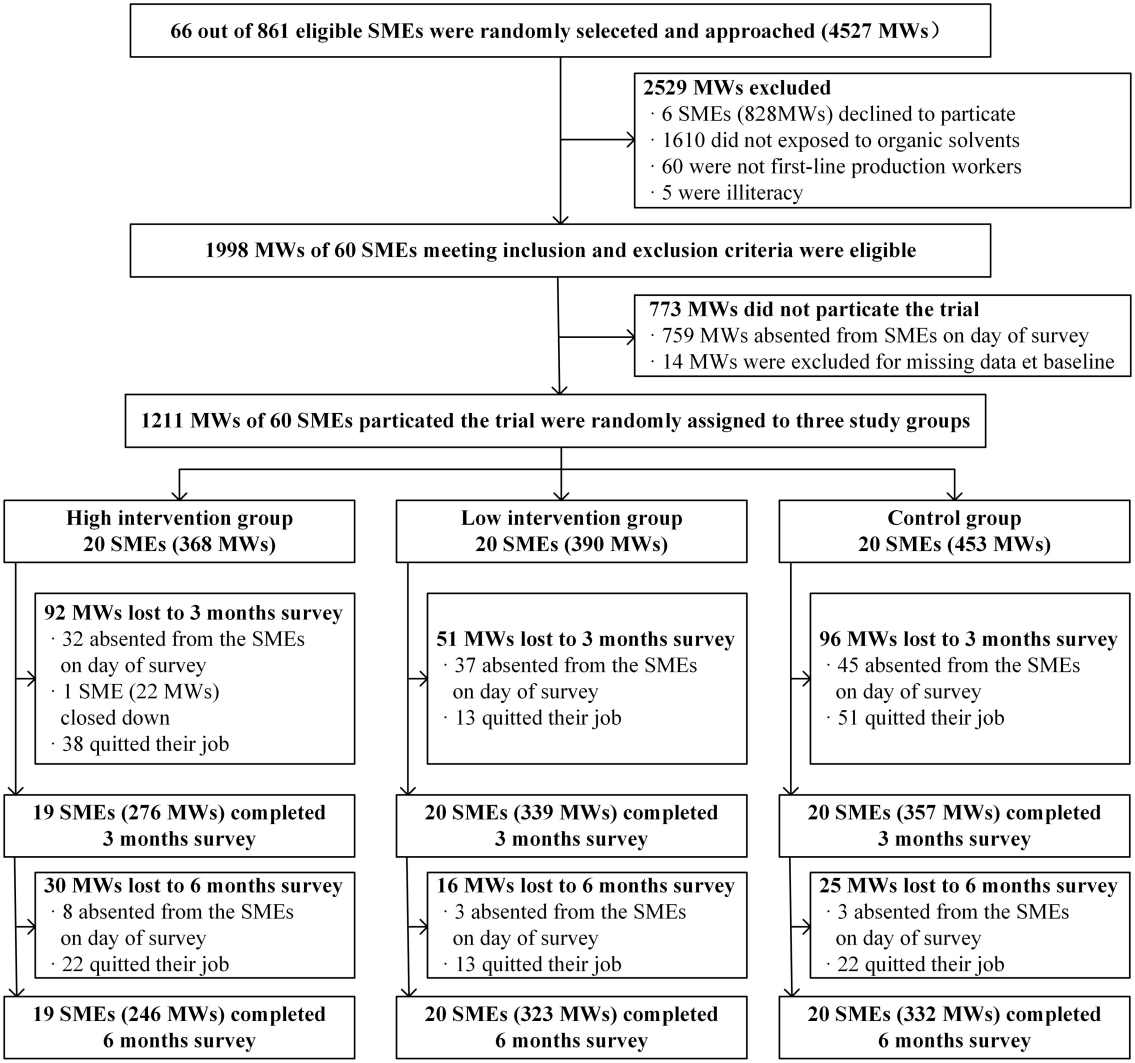
Flow chart of the trial at the enterprise and participant levels. SMEs, small and medium-sized enterprises; IMWs, internal migrant workers.

Figure 1 shows a detailed consort diagram of the trial at enterprise and participant levels. Of the 4,527 individuals assessed for eligibility, 2,529 (55.9%) did not satisfy the inclusion and satisfied exclusion criteria (Figure 1). Of these, 1,610 did not report workplace exposure to organic solvents, 828 declined to participate, 60 were not first-line production workers, and five were illiterate. Of the remaining 1,998 IMWs (44.1%) eligible participants, 759 were absent from work on the day of the survey and 14 were excluded due to missing data at baseline. Therefore, 1,211 participants were enrolled at baseline, with 390 in the low-intensive group, 368 in the high-intensive group and 453 in the control group. The reach rate of all approached potential participants was thus 26.8%, while the participation rate was 60.6% among total eligible individuals. There were 310 (25.6%) migrant workers dropped out at 6-months. Of the remaining 901 (74.4%) participants who completed the trial, 323 were in the low-intensive intervention group, 246 were in the high-intensive intervention group, and 332 were in the control group.

Table 2 shows the baseline characteristics of the migrant workers in each group. The majority of the study participants (71.9%) were male, aged 29–44 years (49.3%), and married (71.9%). Most (68.8%) were educated at or below a high school level. More than half (56.6%) had migrated from a rural area more than 10 years earlier. The average duration of exposure to organic solvents in their current position was 63.6 months (SD = 56.6). The average number of weekly working hours was 55.3 (SD = 9.2).

**Table 2:** Baseline characteristics of participants.

**Characteristics**	**Low-intensive group (*n* = 390)**	**High-intensive group (*n* = 368)**	**Control group (*n* = 453)**	**Total** **(*n* = 1,211)**
**Age (years), mean (SD)** *n* (%) ≤ 28 29–44 45–65	36.3 (9.5) 100 (25.6) 212 (54.4) 78 (20.0)	33.5 (10.8) 146 (39.7) 153 (41.6) 69 (18.8)	34.4 (9.7) 152 (33.6) 232 (51.2) 69 (15.2)	34.8 (10.0) 398 (32.9) 597 (49.3) 216 (17.8)
**Sex**, ***n*** **(%)** Male Female	291 (74.6) 99 (25.4)	245 (66.6) 123 (33.4)	335 (74.0) 118 (26.0)	871 (71.9) 340 (28.1)
**Marriage**, ***n*** **(%)** Married Unmarried	310 (79.5) 80 (20.5)	239 (64.9) 129 (35.1)	322 (71.1) 131 (28.9)	871 (71.9) 340 (28.1)
**Education**, ***n*** **(%)** Primary School Secondary School High School and above	43 (11.0) 236 (60.5) 111 (28.5)	50 (13.6) 197 (53.5) 121 (32.9)	39 (8.6) 268 (59.2) 146 (32.2)	132 (10.9) 701 (57.9) 378 (31.2)
**Duration of migration (years), mean (SD)** ***n*** **(%)** 0–10 > 10	12.4 (7.0) 151 (38.7) 239 (61.3)	10.5 (7.3) 180 (48.9) 188 (51.1)	11.6 (6.8) 195 (43.0) 258 (57.0)	11.5 (7.0) 526 (43.4) 685 (56.6)
Duration of employment at current position (months), mean (SD)	67.0 (50.2)	59.0 (56.7)	64.5 (61.2)	63.6 (56.6)
Duration of employment at current workplace (months), mean (SD)	52.5 (43.2)	31.2 (35.8)	42.4 (48.5)	42.2 (44.0)
Weekly working hours, mean (SD)	55.1 (9.0)	55.2 (8.7)	55.6 (9.7)	55.3 (9.2)
Interpersonal support scores*, mean (SD)	10.0 (3.3)	9.8 (3.7)	10.0 (3.6)	9.9 (3.5)
Social model scores*, mean (SD)	7.8 (2.4)	7.9 (2.5)	7.8 (2.6)	7.8 (2.5)

SD, standard deviation; The symbol * provided in table means Interpersonal support scores included three questions and social model scores included two questions. The score range of interpersonal support and social models was 3–15 and 2–10, respectively, with higher scores indicating stronger support or more positive models.

### Adoption

According to the enterprise information provided by Guangzhou Baiyun District Administration of Work Safety, a total of 861 SMEs were eligible for the trial. We contacted 66 SMEs and sent an informed consent form outlining the purpose, interventions, and main contents of the questionnaires to the OSH managers of each enterprise, who were given 1 week to decide to participate in this study. Six of the 66 SMEs declined, and 60 agreed to participate, with an overall response rate of 90.9% (Figure 1). Table 3 presents an overview of the baseline characteristics of the 60 participating enterprises. The average scale was 125 IMWs (SD = 118), and the enterprises belonged to various organic solvent industries, including furniture, leather goods, and electronics manufacturing, paints and coatings, and plastic and plastic cement. Prior to random allocation, all 60 enterprises had generally established OSH practices and policies for the routine supervision and management of PPE utilization. However, six (10%) enterprises did not offer regular occupational health check-ups for their workers, which we emphasized should be improved upon.

**Table 3:** Baseline characteristics of the 60 study SMEs.

**Characteristics**	**Low-intensive group (*n* = 20)**	**High-intensive group (*n* = 20)**	**Control group (*n* = 20)**	**Total** **(*n* = 60)**
**Industry (** ***n*** **)**				
Furniture Leather goods Electronic manufacturing Paints and coatings Plastic and plastic cement Others Enterprise scale (IMWs), mean (SD) Provide occupational safety and health training (*n*) Provide free PPE to employees (*n*) Instruct employees in PPE utilization regularly (*n*) Provide regular supervision on PPE utilization (*n*) Offer occupational health check-ups (*n*)	8 2 2 3 2 3 146 (124) 20 20 19 20 17	9 1 2 2 2 4 88 (81) 20 20 20 20 18	6 4 3 3 2 2 142 (137) 20 20 20 20 19	23 7 7 8 6 9 125 (118) 60 60 59 60 54

*SD, standard deviation; PPE, personal protective equipment*.

At the intervention deliverer level, 19 staff—eight local government staff in charge of OSH and 11 investigators of our research team—coordinated the implementation of the intervention. Meanwhile, 25 IMWs volunteered as peer-leaders to deliver the peer education sessions.

### Implementation

Prior to the interventions, investigators were trained by the principal investigator of this study and experts from the Guangdong Prevention and Treatment Center for Occupational Diseases in terms of intervention contents, investigation skills, quality control and trial ethics to ensure that the multi-component interventions were implemented as intended. Neither major problems nor adverse incidents occurred during the intervention.

As seen in Table 4, all 40 (100.0%) SMEs in the intervention groups exhibited great fidelity and compliance with the delivery of traditional occupational health education and mHealth interventions. One enterprise in the high-intensive intervention group failed to provide interventions to the IMWs as planned because it ceased trading shortly after this study was launched. However, the fidelity of peer education in the high-intensive intervention group was not adequate. Only five (20.0%) of the 25 peer leaders organized required six peer education sessions during the study period, whereas three (12.0%) did not provide a single peer education session. The specific methods of, and barriers to peer education process are outlined in Table 4. Slightly more than half (52.0%) of the peer leaders conducted peer education sessions during meals or during breaks by demonstrating how to wear RPE. 32.0% of peer leaders complained that scheduling difficulties hindered delivery of the peer education sessions, while 28% stated that they were unfamiliar with some of the peer group members.

**Table 4:** Implementation results of three intervention elements at enterprise and peer-leader levels.

**Indicators**	***n***	**%**
**Traditional occupational health education delivery at enterprise level (*****N*** **=** **40 SMEs)**		
Occupational health lectures delivered Brochures delivered twice (baseline and 3-months) Posters hung in the workspace at every enterprise	40 40 40	100.0 100.0 100.0
**mHealth intervention delivery at enterprise level (*****N*** **=** **40 SMEs)**		
mHealth information provided twice per week *via* instant message apps	39	97.5
**Peer education implementation at peer-leader level (*****N*** **=** **25 peer education groups)**		
**Number of peer education sessions organized**		
0 session 1–3 sessions 4–5 sessions 6 sessions	3 6 11 5	12.0 24.0 44.0 20.0
**Methods of delivering the peer education sessions**		
Gather group members together to communicate with each other Organize group members to learn occupational health-related books Show how to wear PPE Organize group members to watch occupational health videos Oral explanation during meal or during breaks	12 11 13 2 13	48.0 44.0 52.0 8.0 52.0
**Barriers to deliver the peer education sessions**		
Group members were unwilling to participate Limited interpersonal abilities to organize group activities Inconvenient time of group members Unfamiliar with group members Communication barrier with group members	4 4 8 7 2	16.0 16.0 32.0 28.0 10.0

As shown in Table 5, the participants' compliance with traditional health education was higher than the compliance with mHealth intervention in both the low- and high-intensive intervention groups. All IMWs participated in occupational health lectures and received brochures. Compared with the low-intensive group, IMWs in the high-intensive group had a higher rate of compliance with the mHealth intervention (e.g., the percentage of IMWs who read the messages provided *via* instant message apps in the low- and high-intensive group was 65.0% vs. 78.5%, *p* < 0.001). Of the high-intensive intervention participants, 92.7% (238/246) reported that they had attended at least one peer education session, while only 31.7% (78/246) stated that they had taken part in four or more peer education sessions, giving it the lowest reception rate of the three intervention components. When asked about their satisfaction with the program, 9.1%, 36.6%, and 50.1% of participants stated that the program had been “very useful”, “useful” or “somewhat useful”, respectively, whereas 4.2% felt that the program was not useful to them.

**Table 5:** Compliance results of IMWs in two intervention groups at participant level (*n* / %).

**Intervention component**	**Compliance**	**Low-intensive**	**High-intensive group**	**χ^2^**	***P***
		**group (*n* = 323)**	**(*n* = 246)**		
**Traditional occupational health education**					
Browse occupational health-related posters	Yes No	215 (78.8) 58 (21.2)	158 (78.2) 44 (21.8)	0.02	0.888
Participate in occupational health lectures	Yes No	323 (100.0) 0	246 (100.0) 0	-	-
**mHealth intervention**					
Follow the WeChat official account of the program	Yes No	147 (53.8) 126 (46.2)	155 (74.9) 52 (25.1)	22.32	**<0.001**
Join the WeChat or QQ groups of the program	Yes No	114 (41.8) 159 (58.2)	146 (70.9) 60 (29.1)	40.11	**<0.001**
Read the messages provided *via* the instant message apps	Yes No	180 (65.0) 97 (35.0)	164 (78.5) 45 (21.5)	10.48	**<0.001**
**Peer education**					
Number of peer education sessions Attended	No (0) Low (1–3) High (4–6)	-	18 (7.3) 150 (61.0) 78 (31.7)	-	-
**Overall**	Yes No	193 (59.8) 130 (40.2)	159 (64.6) 87 (35.4)	1.41	0.235

*WeChat and QQ are the two most popular instant messaging apps in China. -: No data or no measurement. The bold values provided in table means statistically significant results*.

### Effectiveness

Our team previously published the results on the effectiveness of the intervention with respect to the primary and secondary outcomes (9). Briefly, at 6-months, the rate of appropriately using RPE among IMWs increased by 20.2% in the high-intensive group (54.6% vs. 74.8%) and 12.0% in the low-intensive group (50.5% vs. 62.5%), whereas the rate in the control group (reference) almost remained stable (51.9% vs. 51.2%) (high-intensive intervention: aOR = 2.99, 95% CI: 1.75–5.10, *p* < 0.001; low-intensive intervention: aOR = 1.91, 95% CI: 1.17–3.11, *p* = 0.009). At 6-months, we also noted significant effects of the high-intensive intervention on all secondary outcomes compared with the control group.

**Figure 2:**
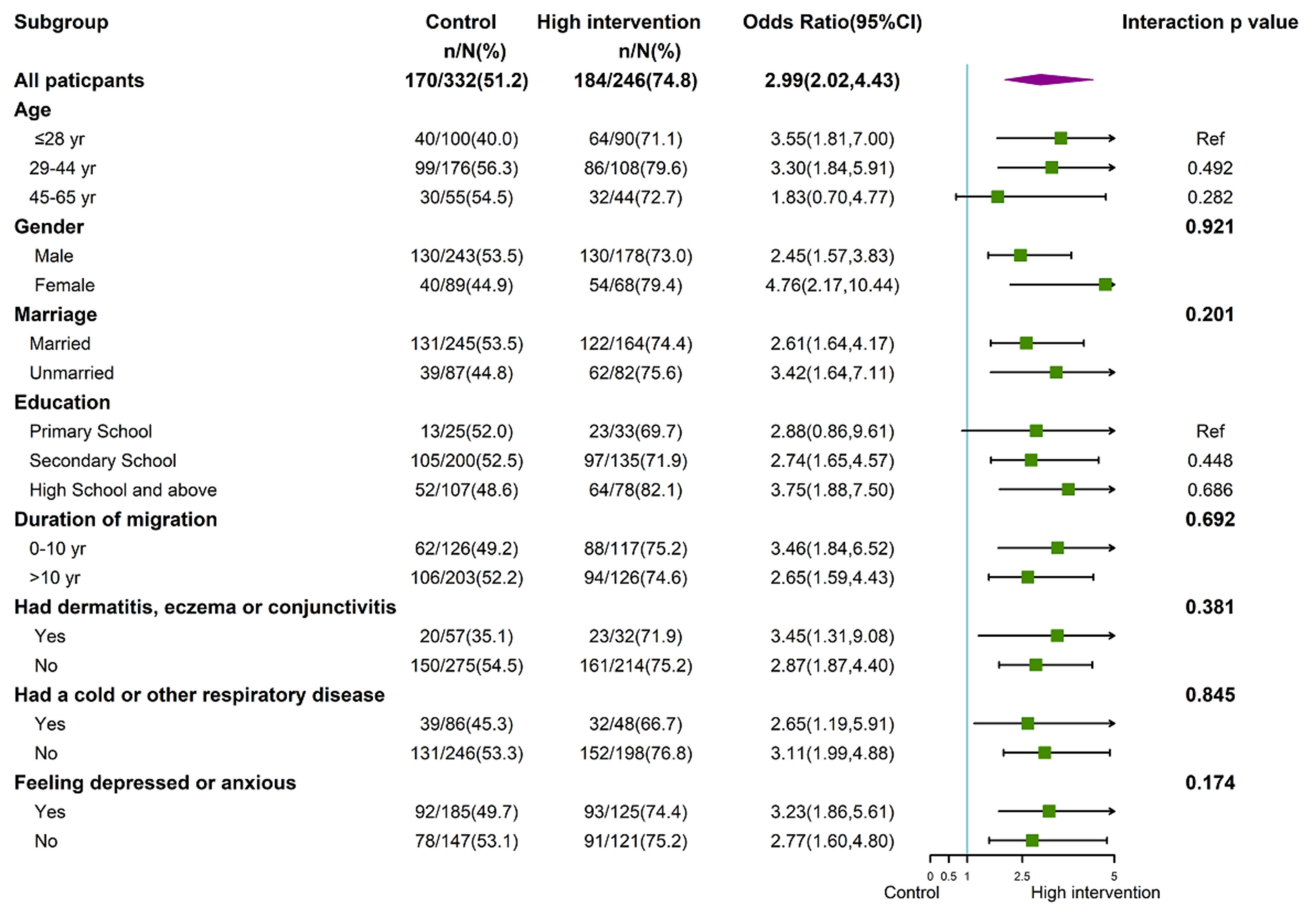
Forest plot of subgroup analyses between the high-intensive intervention and the control groups at 6-months.

Figure 2 shows the results of subgroup analyses comparing the high-intensive intervention group and the control group at 6-months. No statistically significant differences were seen in appropriate RPE use among the different subgroups (*p* > 0.05 for all interactions). In other words, the effect of the interventions on the primary outcome across subgroups of the high-intensive intervention group was robust but not heterogeneous. Subgroup analyses comparing the low-intensive intervention and the control group at 6-months gave similar results (Supplementary Figure 1).

Table 6 shows the impact of intervention compliance on the appropriate use of RPE. At 6-months, IMWs in the two intervention groups who did not comply with the interventions exhibited no statistically significant intervention effects compared with the control group. However, at 6-months, IMWs who complied with the intervention components in both intervention groups were more likely to wear RPE appropriately than those in the control group (low-intensive intervention group: **aOR** **=** **2.58**, 95% CI: 1.56–4.28, *p* < 0.001; high-intensive intervention group: aOR = 7.52, 95% CI: 3.72–15.23, *p* < 0.001).

**Table 6:** Impact of intervention compliance on the use of RPE in the low- and high-intensive intervention groups.

**Intervention component**	**Compliance**	**Low-intensive intervention group**						**High-intensive intervention group**					
		**3-months**			**6-months**			**3-months**			**6-months**		
		***n (%)***	**aOR (95% CI)**	***p***	***n (%)***	**aOR (95% CI)**	***p***	***n (%)***	**aOR (95% CI)**	***P***	***n (%)***	**aOR (95% CI)**	***p***
**Traditional occupational** **health education**													
Browse occupational health-related posters	Yes No	149 (61.9) 28 (57.6)	1.84 (0.91, 3.04) 0.83 (0.37, 1.88)	0.218 0.652	163 (75.8) 29 (59.6)	**2.34 (1.73, 4.88)** 1.43 (0.19, 1.99)	**<** **0.001** 0.453	101 (63.5) 26 (59.1)	1.21 (0.69.2.13) 1.19 (0.47, 3.01)	0.506 0.712	120 (75.9) 30 (68.2)	**2.64 (1.45, 4.80)** 2.01 (0.78, 5.20)	**0.010** 0.150
	Control	191 (53.5)	Ref	-	170 (51.2)	Ref	-	191 (53.5)	Ref	-	170 (51.2)	Ref	-
**mHealth intervention**													
Follow the WeChat official account of the program	Yes No	98 (66.2) 78 (61.9)	1.68 (0.94, 2.99) 1.35 (0.74, 2.46)	0.083 0.331	105 (71.4) 74 (58.7)	**2.40 (1.34, 4.33)** 1.26 (0.69, 2.29)	**<** **0.001** 0.462	103 (66.0) 28 (53.8)	1.21 (0.69, 2.13) 1.39 (0.58, 3.32)	0.510 0.459	119 (76.8) 34 (65.4)	**2.91 (1.41, 6.02)** 2.22 (0.73, 6.72)	**<** **0.001** 0.161
	Control	191 (53.5)	Ref	-	170 (51.2)	Ref	-	191 (53.5)	Ref	-	170 (51.2)	Ref	-
Join the WeChat or QQ groups of the program	Yes No	73 (63.5) 104 (65.4)	1.35 (0.72, 2.54) 1.69 (0.97, 2.94)	0.352 0.074	81 (71.1) 98 (61.6)	**2.18 (1.14, 4.16)** 1.52 (0.88, 2.65)	**0.024** 0.135	94 (63.9) 36 (60.0)	1.15 (0.64, 2.04) 1.55 (0.68, 3.50)	0.643 0.293	109 (74.7) 43 (61.7)	**2.66 (1.27, 5.56)** 2.48 (0.61, 4.22)	**0.013** 0.154
	Control	191 (53.5)	Ref	-	170 (51.2)	Ref	-	191 (53.5)	Ref	-	170 (51.2)	Ref	-
Read the messages provided via the instant message apps	Yes No	116 (64.1) 65 (67.0)	1.35 (0.83, 2.21) 1.12 (0.28, 7.62)	0.226 0.111	129 (71.7) 55 (56.7)	**1.94 (1.18, 3.21)** 1.48 (0.63, 3.48)	**0.012** 0.372	108 (65.5) 24 (53.3)	1.43 (0.83, 2.46) 0.64 (0.22, 1.84)	0.192 0.412	124 (75.6) 32 (71.1)	**2.63 (1.49, 4.64)** 2.24 (0.73, 6.84)	**<** **0.001** 0.163
	Control	191 (53.5)	Ref	-	170 (51.2)	Ref	-	191 (53.5)	Ref	-	170 (51.2)	Ref	-
**Peer education**													
Number of peer education sessions attended	No (0) Low (1–3) High (4–6)	**-**	**-**	**-**	**-**	**-**	**-**	10 (55.6) 88 (54.7) 72 (74.2)	3.30 (0.89, 12.25) 0.75 (0.43, 1.29) **3.03 (1.49, 6.17)**	0.618 0.290 **0.002**	11 (61.1) 100 (66.7) 71 (91.0)	2.93 (0.56, 6.71) 1.45 (0.82, 2.57) **16.59 (6.15, 44.76)**	0.074 0.207 **<** **0.001**
	Control							191 (53.5)	Ref	-	170 (51.2)	Ref	-
**Overall**	Yes No	130 (67.0) 77 (53.1)	1.96 (0.75, 3.22) 1.48 (0.86, 2.40)	0.107 0.172	140 (72.5) 62 (57.7)	**2.58 (1.56, 4.28)** 1.45 (0.85, 2.47)	**<** **0.001** 0.171	103 (64.4) 67 (57.8)	1.19 (0.69,2 .05) 1.63 (0.93, 2.84)	0.531 0.087	117 (73.6) 67 (64.8)	**7.52 (3.72, 15.23)** 1.94 (0.70, 3.40)	**<** **0.001** 0.121
	Control	191 (53.5)	Ref	-	170 (51.2)	Ref	-	191 (53.5)	Ref	-	170 (51.2)	Ref	-

*n (%): The frequencies and percentages of IMWs using RPE appropriately. aOR, adjusted odds ratio; CI, confidence interval; Ref, reference. Control: Control group. -: No data or no measurement. aORs and 95% CIs were generated from generalized linear mixed models by adjusting for age, gender, marital status, education, duration of migration, duration of employment at current position, duration of employment at current workplace, weekly working hours, interpersonal influence, enterprise scale, and occupational health service provision of enterprises. Bold figures: Statistically significant results*.

Specifically, the rate of appropriate RPE use among IMWs who complied with the traditional occupational health education in the low- and high-intensive intervention groups was higher than that of the control group (75.8% and 75.9% vs. 51.2%; low-intensive intervention group: aOR = 2.34, 95% CI: 1.73–4.88; high-intensive intervention group: aOR = 2.64, 95% CI: 1.45–4.80). Improved RPE use was also seen among IMWs who complied with the mHealth intervention in both intervention groups compared with the control group (e.g., IMWs who read the messages provided *via* the instant message apps in the low- and high-intensive intervention groups were more likely to use RPE appropriately compared with the control group: 71.7% and 75.6% vs. 51.2%; low-intensive intervention group: aOR = 1.94, 95% CI: 1.18–3.21; high-intensive intervention group: aOR = 2.63, 95% CI: 1.49–4.64). IMWs who attended four to six peer education sessions in the high-intensive intervention group were more likely to wear RPE appropriately than those in the control group (91.0% vs. 51.2%, aOR = 16.59, 95% CI: 6.15–44.76). However, no significant effects were seen among IMWs who attended zero to three peer education sessions.

### Maintenance

Of the intervention group participants, 93.1% (448/481) IMWs stated that they would keep wearing their RPE. A majority of peer leaders (20/25) stated that they would continue to remind and supervise their workmates to maintain good practice. Organizations and individuals, including the Administration of Work Safety, SMEs and IMWs, could continue to use the intervention materials free of charge after the intervention, including the occupational health lecture PPT, brochures, posters, mHealth video materials, and peer education manuals, so as to promote future application and transformation of these methods. Subsequently, supported by Guangdong Provincial Health and Family Planning Commission, an occupational health manual designed by the research team was distributed to migrants throughout Guangdong Province at no extra cost.

## Discussion

As far as we are aware, this study contains the first multi-component occupational health behavioral intervention program to integrate traditional education and mHealth interventions with peer education to promote occupational health behavior, knowledge and attitude of IMWs working in SMEs. This paper demonstrated that a detailed implementation evaluation using Glasgow's RE-AIM framework to assess the feasibility, reach, adoption, implementation, and maintenance of a program can assist with the interpretation of program outcomes.

Program reach reflects a willingness to participate in a well-designed program among all potential participants in the target population (18). The individual-level reach rate (26.8%) of this complex intervention conducted was higher than that of other occupational healthcare programs, whose reaches ranged from 2.4 to 25% (25–28). Our good level of participation rate may be due to several factors, including assistance provided by the local administration, a good occupational health atmosphere in enterprises (i.e., the vast majority of research enterprises provided due occupational health services), and the unique recruitment strategy used in this study, whereby we approached the managers first, and then managers invited IMWs. Unlike other methods of recruitment, such as invitation by mail, telephone, and posters, which are less likely to attract the participation of the target groups with a lack of pertinence, direct manager invitation might motivate IMWs to participate (29, 30). The drop-out rate of our program was much lower in comparison with that of another enterprise-based intervention carried out among IMWs in China [25.6% vs. 88.5% (31) at six-months]. Because of high mobility and employment instability of migrant workers, interventions targeting them typically have a higher drop-out rate than the general population (32). In China, most IMWs are moved from rural to urban areas, where they have two peaks of return (33). The biggest returning peak is during the Chinese New Year, which is the most important holiday for family reunions contributing to the high attrition rate of Zhu et al.' s research (31). In contrast, we avoided this peak by completing the last intervention contact before the Chinese New Year in 2016. The main reason for drop-out was that 237 IMWs left their jobs, with an attrition rate of 19.7% during the third months (October), which is the seasonal harvesting peak and another key time for IMWs to return home in China. In future interventions with similar contexts, researchers should consider the inherent characteristics of seasonal migration of IMWs and rationalize the study duration to reduce the drop-out rate of participants.

The adoption results demonstrated a willingness to initiate an intervention at both the setting and intervention deliverer levels. We collaborated with the local Administration of Work Safety in the study district to identify all relevant workplaces. To reduce workload and cost, we did not approach all 861 SMEs identified, as 60 was estimated to be a sufficient sample size (8). Only six SMEs declined to participate, with an overall adoption rate of 90.9%, suggesting that our recruitment strategy was feasible and achievable. At the intervention deliverer level, coordination with the local government and the commitment of local political leaders might have facilitated program uptake. This finding is consistent with Zahra et al.' s finding in India that assistance from local political leaders promoted high adoption rates of interventions (34). These findings revealed that enterprise-based interventions would proceed more smoothly with the engagement of relevant stakeholders.

Examining program implementation was essential part of our study, allowing us to examine program fidelity at the setting level and evaluate the reception of the intervention strategies at the participant level, so that we could determine whether the effectiveness of the intervention was due to successful implementation (35). At the provider level, the estimated fidelity rate, which means the extent to which the interventions were delivered as intended, was 100% for the traditional occupational health education intervention, compared with 97.5% for the mHealth intervention, and 20% for peer education. Since traditional occupational health education was conducted after the baseline survey, it was not surprising to see its successful implementation. The mHealth intervention was delivered by well-trained trainers of the research team, basically ensuring that it was implemented as planned. At the participant level, we assessed participants' satisfaction and compliance with the interventions, which reflects participant's responses and acceptance of interventions (19). Overall, the intervention participants were satisfied with the program and stated that the program could prevent them from contracting severe occupational illnesses. In terms of participants' compliance, IMWs were provided with occupational health lectures and brochures at the very beginning of the intervention and could view the mHealth-related materials in their leisure time, and therefore their compliance with traditional health education and mHealth intervention was higher than compliance with peer education, which might take up a large amount of working time and affect the daily production and incomes of workers.

Considering differences in language, self-identity, and interpersonal communication between internal migrant workers and native workers (36), we incorporated peer education into our occupational health intervention for IMWs. We found that compared with the low-intensive intervention group, the high-intensive intervention group, which received peer education, exhibited not only an improved intervention effect but also better compliance with the mHealth intervention. These findings highlight a significant role of peer education as a means to enhance positive interpersonal relationships and social support between peers, thus improving IMWs' compliance with various interventions (37). However, compared with the other two intervention components, peer education delivered by peer leaders was not found to be beneficial and IMWs' adherence to peer education was relatively low. Only 31.7% of IMWs in the high-intensive intervention group reported attending more than half of designed peer education sessions. In our study, peer leaders were willing to self-nominate for peer leadership, and we could not guarantee that all peer leaders were competent and responsible for peer education implementation. Furthermore, we noted that one-third of these peer leaders reported factors that may have contributed to their poor delivery of peer education sessions, such as scheduling issues, being unfamiliar with their peers, limited interpersonal abilities, and unwillingness of their peers to engage. Therefore, strategies to improve IMWs' compliance with peer education attendance will be vital to the future dissemination of similar interventions. First, detached strategies for peer leader recruitment instead of self-nomination might be more beneficial (38). Peer leaders with outgoing personalities and strong communication skills should be recruited. Second, it will be necessary to strengthen the training, assistance, and supervision of peer leaders to improve their ability and self-efficacy. Online training or other flexible training methods may provide ongoing guidance to peer leaders. Third, in the design of peer sessions, increasing more diversified intervention contents and forms might facilitate interactive communication between peers. Fourth, better monetary or non-monetary incentives to promote enthusiasms and increase attendance could be considered. Finally, inviting OSH-related professionals to assist in implementing peer education sessions may improve workers' adherence (39), however, the feasibility and effectiveness of this approach need to be confirmed in further research.

Maintenance refers to long-term effectiveness at the individual or a sustained impact on routine organizational practices at the setting level at 6 or more months post-intervention (19). As many other trials, we did not collect follow-up data beyond 6 months post-intervention, given the lack of feasibility of a continuous workplace investigation (40, 41). Therefore, the enterprise-level maintenance remains unreported as a limitation of this study. We assessed only the willingness of participants and peer leaders to maintain proper use of RPE and to supervise their workmates in the future, and most of them responded positively, suggesting that our program has the potential to be sustained at the individual level over time.

This study has some limitations. Firstly, some program implementation data, such as the intervention outcomes, participants' compliance with the mHealth intervention, and satisfaction with the program, were self-reported by SMEs and IMWs, therefore, the study was subject to reporting bias and recall bias (42). Secondly, the study settings and participants were limited to one district of Guangzhou city. Hence, the generalizability of our findings should be viewed with caution. Additionally, we did not assess all potential indicators of the RE-AIM framework, such as the reasons for non–participation, the characteristics of non–participants, setting-level maintenance, and cost of the intervention. Finally, we only used quantitative methods in this study. A combined quantitative and qualitative method might provide a deeper understanding of the implementation process and how outcomes arise (43).

## Conclusions

This comprehensive implementation evaluation using the RE-AIM framework demonstrated that the occupational health behavioral intervention program, which combined traditional education with mHealth intervention and peer education, was feasible and acceptable in promoting the use of RPE and related occupational health knowledge and attitude among IMWs in SMEs. Participants' adherence to peer education was a key factor in successful implementation and had a significant impact on program effectiveness, however, the fidelity of and participation in this component were relatively low. Future programs should focus on effective implementation strategies for improving peer education sessions delivery.

## Data Availability Statement

The raw data supporting the conclusions of this article will be made available by the authors, without undue reservation.

## Ethics Statement

The studies involving human participants were reviewed and approved by Research Ethics Committee of the School of Public Health, Sun Yat-sen University. The patients/participants provided their written informed consent to participate in this study.

## Author Contributions

CL led this implementation evaluation including the evaluation design, data analysis, and drafted the initial manuscript. WC (Principal Investigators), LL, and GZ were involved in writing the original study protocol and contributed to the study design and funding acquisition. WC and TL contributed to investigation and data collection. XL contributed to intervention resources provision and technical supports. All authors read and approved the final manuscript. All authors contributed to the article and approved the submitted version.

## Funding

This trial was funded by National Science Foundation of China (81402767), China Medical Board (13–175) and Sun Yat-sen University (15ykpy08). The funders had no involvement in study design, collection, analysis, interpretation of data, writing of the report, and the decision to submit the paper for publication.

## Conflict of Interest

The authors declare that the research was conducted in the absence of any commercial or financial relationships that could be construed as a potential conflict of interest.

## Publisher's Note

All claims expressed in this article are solely those of the authors and do not necessarily represent those of their affiliated organizations, or those of the publisher, the editors and the reviewers. Any product that may be evaluated in this article, or claim that may be made by its manufacturer, is not guaranteed or endorsed by the publisher.

## Abbreviations

IMWs: Internal Migrant Workers
SMEs: Small and Medium-sized Enterprises
RPE: Respiratory Protective Equipment
OSH: Occupational Safety and Health
NHC: National Health Commission
CRCT: Cluster Randomized Controlled Trial
PPE: Personal Protective Equipment
SD: Standard Deviation
GLMMs: Generalized Linear Mixed Models
OR: Odds Ratio
CI: Confidence Interval
aOR: Adjusted Odds Ratio.
