# Supplementary material for: Implementation Evaluation of a Cluster Randomized Controlled Trial to Promote the Use of Respiratory Protective Equipment Among Migrant Workers Exposed to Organic Solvents in Small and Medium-Sized Enterprises
Source: Front Public Health. 2022 Jul 11;10:772632. doi: 10.3389/fpubh.2022.772632 (PMC9319860; doi:10.3389/fpubh.2022.772632)
Supplement: Supplementary file 1 [file Data_Sheet_1.docx]

Supplementary Material

# Supplementary Figures and Tables

## Supplementary Figures


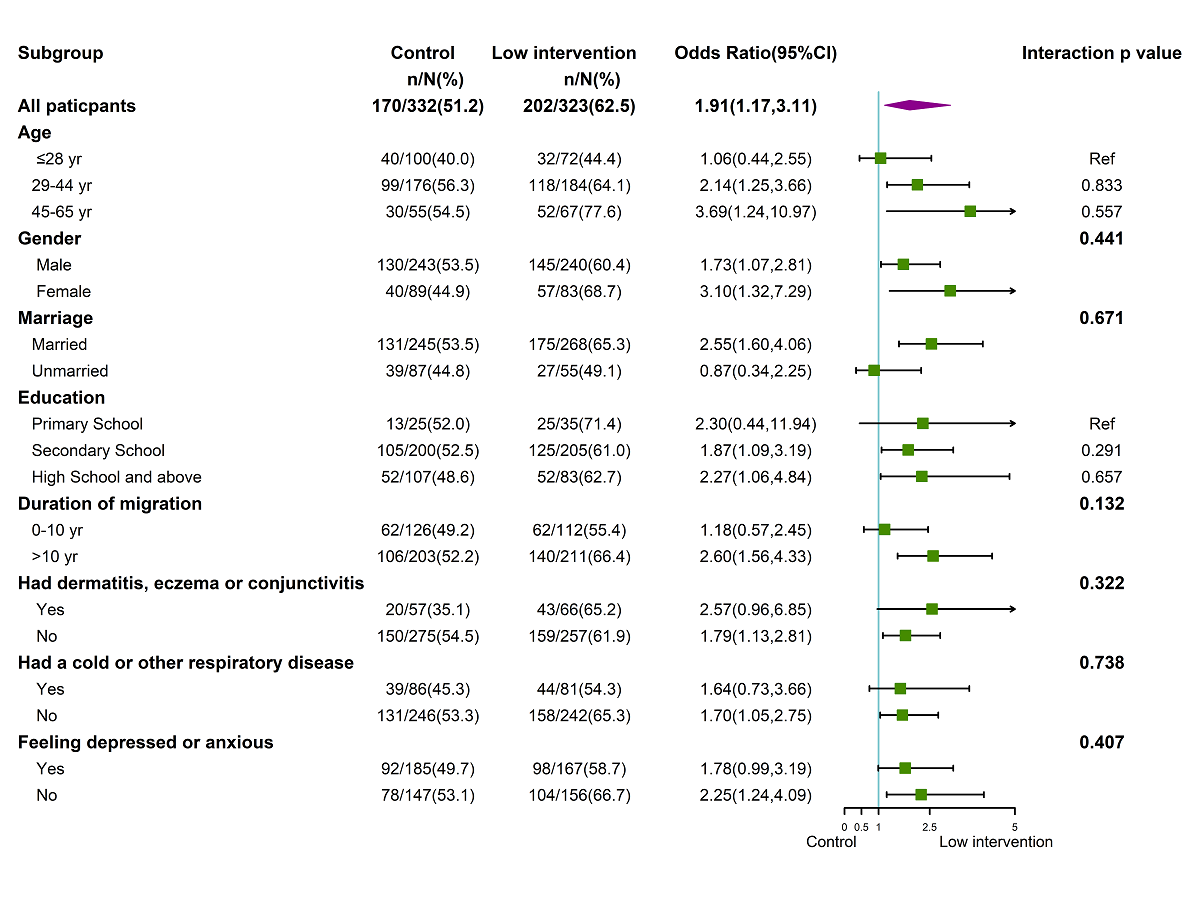


**Supplementary Figure 1.** Forest plot of subgroup analyses between the low-intensive intervention and the control groups at 6-month.

## Supplementary Tables

**Supplementary Table 1. Questions for measuring key outcome variables.**

| **Variables** | **Questions** | **Range** | **Cronbach’ ɑ** |
| --- | --- | --- | --- |
| **Intervention compliance** | Participate in occupational health lectures.  Browse occupational health-related posters designed by our research team.  Join the WeChat or QQ groups of the program.  Follow the WeChat official account of the program.  Read the messages provided via the instant message apps.  Number of peer education sessions attended. | 0–7  1 = yes, 0 = no,  1 = yes, 0 = no,  1 = yes, 0 = no,  1 = yes, 0 = no,  1 = yes, 0 = no,  2= “4–6” , 1= “1–3”, 0 = “0” | 0.82 |
| **Occupational health attitude**  Perceived benefits  Perceived barriers  Self-efficacy | I wear RPE because I may get sick from exposure to organic solvents.  Use RPE during work can reduce exposure to organic solvents.  Appropriate use of RPE could prevent occupational diseases.  I would choose not to use RPE because it is uncomfortable.  I would choose not to use RPE because it affect personal appearance.  I would choose not to use RPE because it lower the work efficiency.  I believe I can properly use RPE when exposed to organic solvents.  I believe I can use RPE at all times when exposed to organic solvents.  I believe I will use RPE if it is available at the enterprise. | 5–45  1 = strongly disagree,  2 = disagree,  3 = don't agree or disagree,  4 = agree,  5 = strongly agree | 0.84 |

| **Occupational health knowledge** | 1. Organic solvents can cause acute and chronic poisoning.  2. Organic solvents can be absorbed into the body through the skin.  3. Before contact with organic solvents, you should read the Material Safety Data Sheet to understand the product composition, toxicity, and safety measures to be followed.  4. You can wash your hands with organic solvents to remove grease.  5. *Kaiyoushui, Baidianyou, Kaijiaoshui, Xibanshui* (Common name for organic solvents products in Chinese) contain substances harmful to health.  6. In case of contact with organic solvents, you can rinse the contaminated skin with water.  7. Personal protective equipment should be worn at all times in environments where organic solvents are present.  8. As long as the protective equipment is not damaged, it can be used during the work.  9. Before wearing a protective mask, you should check how it fits your face. Masks that do  not completely cover your mouth and nose will not provide protection.  10. Personal protective equipment contaminated with organic solvents can be discarded directly. | 0–10  1 = yes, 0 = no  1 = yes, 0 = no  1 = yes, 0 = no  1 = no, 0 = yes  1 = yes, 0 = no  1 = no, 0 = yes  1 = yes, 0 = no  1 = no, 0 = yes  1 = yes, 0 = no  1 = no, 0 = yes | 0.78 |
| --- | --- | --- | --- |
| **Participation in occupational health check-ups** | Whether you have taken part in occupational health check-ups during the past six months | 1 = yes, 0 = no |  |
| **Interpersonal influence**  Interpersonal support  Social model | Do your coworkers encourage you to use PPE?  Do your family members encourage you to use PPE?  Do your supervisors encourage you to use PPE?  How often does my team leader use PPE when exposed to toxic substances?  How often do my workmates use PPE when exposed to toxic substances? | 5–25  1 = never / almost never,  2 = rarely,  3 = sometimes,  4 = often,  5 = always / almost always | 0.80 |
